# Post-treatment strategies for pyrophoric KOH-activated carbon nanofibres†

**DOI:** 10.1039/d3ra07096d

**Published:** 2024-01-25

**Authors:** Tom Fischer, Ansgar Kretzschmar, Victor Selmert, Sven Jovanovic, Hans Kungl, Hermann Tempel, Rüdiger-A. Eichel

**Affiliations:** a Forschungszentrum Jülich GmbH, Institute of Energy and Climate Research (IEK-9) – Fundamental Electrochemistry Jülich 52425 Germany; b RWTH Aachen University, Institute of Physical Chemistry Aachen 52056 Germany

## Abstract

The effect of two atmospheric post-treatment conditions directly after the KOH activation of polyacrylonitrile-based nanofibres is studied in this work. As post-treatment different N_2_ : O_2_ flow conditions, namely high O_2_-flow and low O_2_-flow, are applied and their impact on occurring reactions and carbon nanofibres' properties is studied by thermogravimetric analysis (TGA), differential scanning calorimetry (DSC), scanning electron microscopy (SEM), Raman spectroscopy, elemental analysis and CO_2_ and Ar gas adsorption. At high O_2_-flow conditions a pyrophoric effect was observed on the KOH-activated carbon nanofibers. Based on the obtained results from the TGA and DSC the pyrophoric effect is attributed to the oxidation reactions of metallic potassium formed during the KOH activation process and a consequent carbon combustion reaction. Suppression of this pyrophoric effect is achieved using the low O_2_-flow conditions due to a lower heat formation of the potassium oxidation and the absence of carbon combustion. Compared to the high O_2_-flow samples no partial destruction of the carbon nanofibers is observed in the SEM images. The determination of the adsorption isotherms, the surface area, the pore size distribution and the isosteric enthalpies of adsorption show the superior properties under low O_2_-flow conditions. The present micropore volume is increased from 0.424 cm^3^ g^−1^ at high O_2_-flow to 0.806 cm^3^ g^−1^ for low O_2_-flow samples, resulting in an increase of CO_2_ adsorption capacity of 38% up to 6.6 mmol g^−1^ at 1 bar. This significant improvement clearly points out the importance of considering highly exothermic potassium oxidation reactions and possible post-treatment strategies when applying KOH activation to electrospun carbon nanofiber materials.

## Abstract 2

The effect of two atmospheric post-treatment conditions directly after the KOH activation of polyacrylonitrile-based carbon nanofibres is studied in this work. Vigorous potassium oxidation reactions could be avoided by a low O_2_-flow treatment.

## Graphical abstract

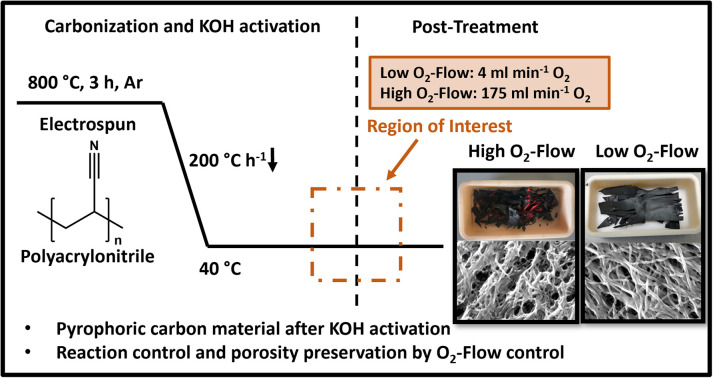

## 1. Introduction

Carbon-based materials are widely applied in research and industry, *e.g.* as a component of devices for energy storage or as adsorbent in gas separation processes.^1–6^ Amongst others, activated carbons are a frequently used material class due to their high micropore volume and high specific surface area. They are synthesized *via* a two-step process, comprised of carbonisation and activation. Typically, the precursor is first carbonised and subsequently activated, but also simultaneous carbonisation and activation processes are described.^7–9^ The applied process and activation method depends on the desired surface morphology and porosity of the carbon for the individual application. Therefore, a variety of activation processes have been developed to optimise the pore structure of carbons, which can be divided into physical activation and chemical activation methods. Physical activation is done using oxidising gases, *e.g.* CO_2_ or H_2_O, whereas chemical activation uses solid activating agents, *e.g.* KOH or H_3_PO_4_ to introduce porosity into the material.^10–12^ As a chemical activating agent for carbon-based materials, KOH is well-established and was patented by Wennerberg *et al.* in 1978.^13^ Since then, KOH activation has been widely used to activate many different precursor materials, *e.g.* coals, biomass and carbon fibres.^1,12,14^ KOH is mainly applied to obtain a high micropore volume and a high specific surface area of the activated materials. Frequently reported BET areas for KOH activated carbon fibres range from 1000 up to 3000 m^2^ g^−1^ and the total pore volume easily reaches up to 2 cm^3^ g^−1^.^9,15^ Despite the widespread application for decades, the exact mechanism of the KOH activation is still discussed. As overall reaction for the KOH activation, eqn (1) is proposed.^1,15–17^

1 6KOH + 2C ↔ 2K + 3H_2_ + 2K_2_CO_3_, Δ*H*_R_ = +428 kJ mol^−1^

Nevertheless, the actual occurring reactions are a sequence of several parallel and consecutive reactions and highly depend on the used precursor.^1,10,18–20^ The dehydration of KOH resulting in K_2_O and H_2_O (eqn (2)) is proposed as starting reaction for the KOH activation of petroleum coke.^15,16^ On elevating the temperature, the carbon and the H_2_O are transformed into H_2_ and CO *via* a coal gasification reaction (eqn (3)). Additional H_2_O reacts *via* the water gas shift reaction with the formed CO resulting in H_2_ and CO_2_ (eqn (4)). The obtained K_2_O (eqn (2)) and CO_2_ (eqn (4)) are transformed to K_2_CO_3_ at temperatures between 400 and 800 °C (eqn (5)). Furthermore, at reaction temperatures >700 °C K_2_O is reduced by H_2_ and carbon to metallic potassium (eqn (6) and (7)), which can intercalate into the carbon lattice.^16^

2 2KOH ↔ K_2_O + H_2_O, Δ*H*_R_ = +245 kJ mol^−1^

3 C + H_2_O ↔ H_2_ + CO, Δ*H*_R_ = +131 kJ mol^−1^

4 CO + H_2_O ↔ H_2_ + CO_2_, Δ*H*_R_ = −41 kJ mol^−1^

5 K_2_O + CO_2_ ↔ K_2_CO_3_, Δ*H*_R_ = −393 kJ mol^−1^

6 K_2_O + H_2_ ↔ 2K + H_2_O, Δ*H*_R_ = +299 kJ mol^−1^

7 K_2_O + C ↔ 2K + CO, Δ*H*_R_ = +430 kJ mol^−1^

Similar reactions were proposed for the KOH activation of multi-walled carbon nanotubes with the formation of K_2_CO_3_*via* redox reactions starting at 400 °C.^17^ In subsequent reactions the K_2_CO_3_ etches the carbon framework and K_2_O and CO are formed (eqn (8)).

8 K_2_CO_3_ + C ↔ K_2_O + 2CO, Δ*H*_R_ = +565 kJ mol^−1^

Similar to the activation reactions on petroleum coke the formation of elemental potassium is described for temperatures higher than 700 °C (eqn (7)). Summarising the proposed reactions involved in KOH activation it can be agreed on three types of pore-forming reactions:

(a) Etching of the carbon material *via* redox reactions (eqn (3), (7), and (8))

(b) Pore formation through gasification, *i.e.* H_2_O, CO, CO_2_, H_2_ (eqn (2)–(4), (6)–(8))

(c) Intercalation of elemental potassium (eqn (6) and (7)) into the carbon lattice, resulting in expansion of the carbon lattice.

Nevertheless, the actual reaction pathway highly depends on the applied activation parameters and the used precursor. Moreover, the structure of the precursor also affects the possibility of potassium intercalation. Overall, these impact factors render the assessment of the exact occurring reaction mechanisms a difficult task.^1,20^

Since the first patents, countless studies have been published on the impact of the different activation parameters, *e.g.* activation temperature, KOH : precursor ratio and activation duration.^20,21^ In contrast, the impact of the atmospheric post-treatment conditions directly subsequent to KOH activation has not been investigated so far. However, this factor may have a serious impact on the obtained activated carbon due to the high reactivity of elemental potassium formed during KOH activation. Elemental potassium is known to react vigorously in the presence of oxygen and water due to the formation of potassium oxides according to eqn (9)–(11).^22,23^9
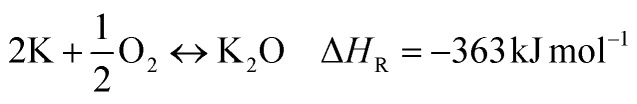

10 2K + O_2_ ↔ K_2_O_2_, Δ*H*_R_ = −496 kJ mol^−1^

11 K + O_2_ ↔ KO_2_, Δ*H*_R_ = −285 kJ mol^−1^

The occurrence of such reactions in potassium-treated carbon materials has been frequently reported and was first observed by Fredenhagen and Cadenbach, who obtained pyrophoric potassium-graphite intercalation compounds in 1926.^24,25^ Additionally, a similar pyrophoric effect is described for the intercalation products of coals and amorphous carbons with K_2_CO_3_ during K_2_CO_3_-catalyzed gasification.^26^ Such an intercalation of potassium from melts into graphite and carbons was detailed studied in several publications.^25,27,28^

The present work investigates, the occurrence of such a pyrophoric effect after KOH activation by application of two different atmospheric post-treatments. Therefore, electrospun PAN-based carbon nanofibres are used, which exhibited carbonisation temperature dependent molecular sieve properties and a remarkable CO_2_/N_2_ adsorption selectivity in previous works.^29–34^ TGA-MS, DSC, SEM, elemental analysis, Raman spectroscopy and gas adsorption techniques are used to investigated occurring reactions and changes to the fibre morphology and porosity.

## 2. Experimental

### 2.1 Carbon nanofibres synthesis

For the synthesis of the carbon nanofibres, solutions of 10 wt.% PAN (150 000 g mol^−1^, BOC Science, USA) in *N*,*N*-dimethyl formamide (VWR Chemicals, Germany) were prepared. All chemicals were used as received without further purification. To obtain a complete dissolution the mixtures were stirred for 48 h at room temperature. Subsequently, the solution was electrospun using an electrospinning device (IME Technologies, Netherlands). The polymer solution was supplied at a flow rate of 120 μL min^−1^ through a 4-tip spinning needle. The spinneret was moved laterally to the collector drum with a speed of 20 mm s^−1^ and a turn delay of 500 ms within a range of 120 mm. The spinning process was conducted at constant climate conditions of 25 °C and 30% relative humidity and the applied voltage was set to 25 kV. The tip-collector distance was 120 mm. The fibres were collected on a rotating drum with a diameter of 90 mm and a rotational speed of 1000 rpm. In total, the spinning was conducted for 3 h, which corresponds to 21.6 mL used spinning solution. Subsequently, oxidative stabilization at 250 °C in air for 15 h was performed at a heating rate of 5 K min^−1^ using a drying cabinet (Binder GmbH, Germany). In the following step, 200 mg of the stabilized nanofibres were impregnated with 10 mL aqueous KOH (*c* = 0.26 mol L^−1^, KOH : PAN weight ratio 3 : 4) for 2 h. Afterwards, the sample was dried at 85 °C for 3 h to remove H_2_O.

The entire carbonisation & activation process was conducted inside a thermogravimetric analyser (STA 449 F1 Jupiter, Netzsch GmbH, Germany) coupled to a mass spectrometer (QMS 403 D Aëlos, Netzsch GmbH, Germany) (TGA-MS). 200 mg of the obtained KOH impregnated nanofibres were transferred into the 5 mL TGA beaker to conduct the simultaneous carbonisation and activation. The TGA furnace was purged trifold with Ar to ensure an inert carbonisation atmosphere. Afterwards, the samples were heated at a rate of 300 K h^−1^ up to 800 °C and held for 3 h at this temperature in inert atmosphere at a flow rate of 40 mL min^−1^ Ar 5.2 (Air Liquide, France). Afterwards, the samples were cooled down to 40 °C at a rate of 200 K h^−1^. Once a temperature of 40 °C was reached, the atmosphere was changed to an O_2_ : N_2_ atmosphere. The used O_2_-flow rates were 4 mL min^−1^ for low O_2_-flow conditions and 175 mL min^−1^ for high O_2_-flow conditions. The respective N_2_ flow rates were 16 mL min^−1^ for low O_2_-flow conditions and 75 mL min^−1^ for high O_2_-flow conditions. After the switch to O_2_ : N_2_ atmosphere, the samples were kept for 45 minutes under the applied atmospheric conditions. After this process steps, the carbon nanofibres were obtained and neutralized using distilled water in several washing steps until a neutral pH value was achieved. Finally, the samples were dried at 100 °C. For each post-treatment, a fivefold determination was carried out.

Additionally, similar experiments were conducted in a horizontal tube furnace (REST-E 400/6, Carbolite Gero GmbH & Co. KG, Germany). 200 mg of the KOH impregnated sample were transferred into an alumina boat and the furnace was trifold purged with Ar. Subsequently, the sample was heated up to 800 °C at a heating rate of 300 K h^−1^ in inert atmosphere at a flow rate of 105 L h^−1^ Ar 5.2 (Air Liquide, France). The temperature was maintained for 3 h and afterwards cooled down at a rate of 200 K h^−1^. At a temperature of 40 °C the furnace was opened and the samples were exposed directly to ambient air, which equals high O_2_-flow conditions.

### 2.2 Material characterization

Mass and temperature changes, as well as the gaseous reaction products were detected by the TGA-MS system. The MS measurements were conducted in multiple ion detection (MID) mode. Relevant gases were identified with a scan measurement for *m*/*z* 0–100 in an additional run prior to the actual measurements. The obtained MID runs were normalized to a reference run, to correct contamination of the device caused by the formation of soot particles during the reaction.

For data evaluation, the peak area for the detected components was determined by integration. The obtained weight normalized peak area is converted into the molar amount of CO_2_ based on a calibration using CaC_2_O_4_ × H_2_O. For the calibration masses of 50, 75, 100, 125 and 150 mg of CaC_2_O_4_ × H_2_O were transferred into the TGA crucible and afterwards heated to 1000 °C. Based on the obtained results, a relation of peak area CO_2_ to molar amount could be drawn (S1).† ^35^

Additionally, differential scanning calorimetry (DSC) measurements were performed on a STA 449 F1 Jupiter (Netzsch GmbH, Germany) to measure the released reaction heats. Prior to the measurements, a calibration using a sapphire disc was performed. The DSC measurements were conducted with an equal heat treatment as for the carbonisation and activation process in the TGA beaker. The sample mass of KOH impregnated nanofibres for these measurements was 20–30 mg. For each post-treatment condition, a triple determination was conducted.

Elemental analysis was conducted using a varioELcube elemental analyser (Elementar, Germany). A triple determination with 2 mg for each sample was carried out to determine C, H and N content. The O content was determined as the difference between the CHN content to the total composition.

Inductively coupled plasma optical emission spectroscopy (ICP-OES) was performed on iCAP 7600 analyser (ThermoFisher Scientific, USA). Two parallel digestions of 100 mg sample each were prepared in a furnace with 250 mg lithium borate. The samples were heated to 1050 °C during 3 hours and maintained for 30 minutes at this temperature. The obtained melt was diluted in 50 mL 5% HNO_3_ and filled up to 100 mL.

A Quanta FEG 650 microscope (FEI, USA) was used to conduct the scanning electron microscopy (SEM) investigations. For image recording an acceleration voltage of 20 kV and an Everhart–Thornley detector was used. Small parts of the sample were fixed on the sample holders using copper strips.

Gas adsorption measurements were performed on a 3P micro 300 (3P Instruments, Germany). Argon (5.2, Air Liquide, France) adsorption measurements were conducted at 87 K and CO_2_ (4.5, Air Liquide, France) adsorption measurements at 273 K. Prior to the measurements the samples were outgassed at 150 °C for 12 h.

The obtained data was evaluated using Asiqwin 5.0 (Quantachrome Instruments, USA). The adsorption isotherms were fitted as a Tóth isotherm^36^ to calculate a mean isotherm for each parameter set. As error assessment the standard deviation was determined. A quenched solid state DFT (QSDFT) equilibrium model was applied to calculate the pore size distribution of the Argon adsorption data using a slit pore model on carbon. The pore size distribution of the CO_2_ adsorption isotherms was obtained by a Monte-Carlo model on carbon for slit pores.

Similar to the isotherm fits a mean value was calculated and the error indicated by the standard deviation. Additionally, the determination of the BET area, taking into account its limitations for microporous materials, was performed.^37,38^

Determination of the adsorption kinetics was performed using an Autosorb iQ 2 (Quantachrome, USA) by measurement of a single point isotherm at 50 mbar at 298 K using the VectorDose™ mode.

For calculation of the isosteric enthalpy of adsorption, CO_2_ adsorption isotherms were measured at 273 K, 283 K and 293 K on a QuadraSorb EVO (Quantachrome, USA) and fitted using a Tóth Fit. The calculation of the isosteric enthalpies of adsorption was conducted *via* the Clausius–Clapeyron approach.^39^

## 3. Results and discussion

**Fig. 1:**
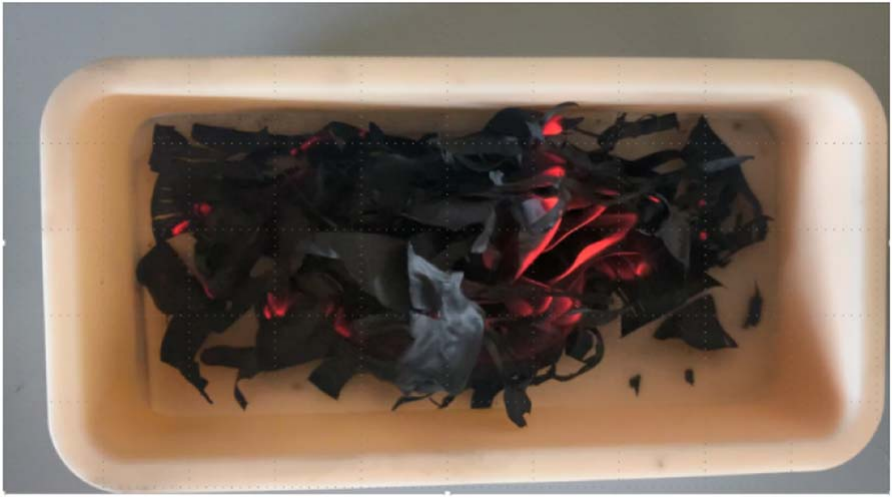
Pyrophoric KOH-activated electrospun PAN-based carbon nanofibres during exposure to ambient air directly after the simultaneous carbonisation and KOH activation. A red glowing of the carbon nanofibres can be observed (a Video of the glowing is attached as ESI†).

In preliminary experiments KOH-activated carbon nanofibers were post-treated at ambient air as it is the usual procedure.^1,11,12^ During the exposure to ambient air, a strong red glowing of the carbon nanofibres was observed, which indicates vigorous potassium oxidation reactions (Fig. 1 and Video in the ESI†).

**Fig. 2:**
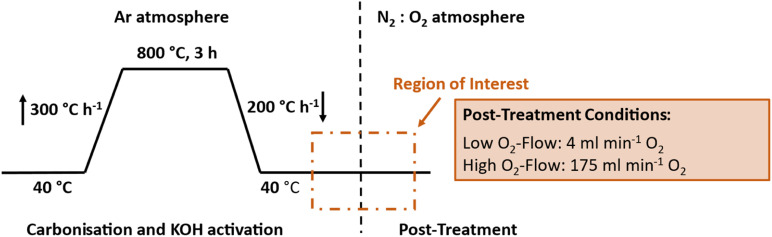
Temperature profile of the applied simultaneous carbonisation and activation in Ar with subsequent post-treatment at 4 mL min^−1^ (low O_2_-flow) and 175 mL min^−1^ O_2_-flow (high O_2_-flow). The vertical dashed line indicates the switch from Ar to N_2_ : O_2_ atmosphere.

Detailed investigations on this pyrophoric effect using a TGA-MS following the heat treatment in Fig. 2 were conducted to get a deeper insight into the occurring reactions. Therefore, two different post-treatment conditions were applied subsequent to the simultaneous carbonisation and activation: one, labelled as ‘low O_2_-flow’ at an O_2_-flow rate of 4 mL min^−1^ (N_2_ : O_2_ 80 : 20) and a second one at 175 mL min^−1^ (N_2_ : O_2_ 30 : 70), denoted as ‘high O_2_-flow’. Additionally, SEM, DSC, elemental analysis, Raman spectroscopy and gas adsorption were used to obtain detailed information on the observed glowing, the reaction process and its impact on the fibre morphology.

### 3.1 High O_2_-flow

**Fig. 3:**
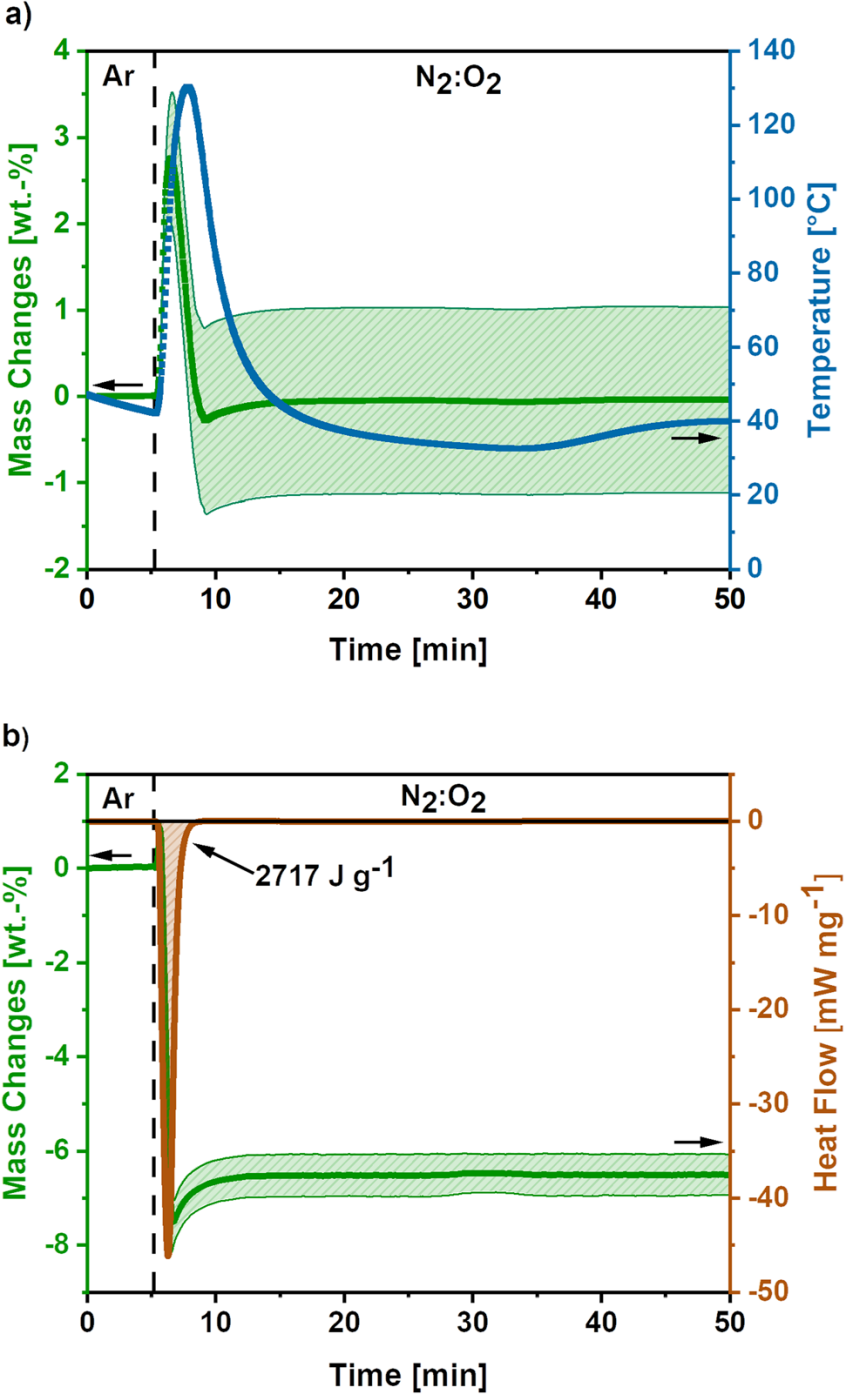
(a) Mass and temperature changes during post-treatment at high O_2_-flow conditions (175 mL min^−1^ O_2_). Average value of 5 measurements. The shaded areas show the standard deviation for the mass signal and the dashed line indicates the switch from Ar to N_2_ : O_2_ atmosphere. (b) DSC-TGA data during high O_2_-flow post-treatment of KOH-activated carbon nanofibres (O_2_-flow 175 mL min^−1^), where the dashed line indicates the atmosphere switch. A release of −2717 J g^−1^ reaction heat accompanied by a mass loss 8.2 wt.% is observed.

In Fig. 3a the mass and temperature changes of the freshly activated samples on switching the atmosphere from Ar to a mixture of N_2_ : O_2_ (30 : 70) are shown for the high O_2_-flow. The vertical dashed line indicates the switch from Argon to N_2_ : O_2_ (30 : 70) atmosphere at 175 mL min^−1^ O_2_-flow. Almost instantly after the change in atmosphere, a mass gain of 2.8 ± 0.7 wt.% is observed. Afterwards, a mass loss of 3.1 ± 1.1% occurs before the mass slightly increases again. Simultaneously, the temperature rises to 130 °C and cools down again to the temperature set point of 40 °C afterwards. However, it must be emphasized, that this is only an apparent temperature, as the reaction heat is partially consumed by the heat capacity of comparatively large TGA crucible. The actual temperature of the sample during the reaction is significantly higher.

The mass gain and the temperature rise can be assigned to the oxidation reactions of elemental potassium, which was formed during the KOH activation reactions (eqn (6) and (7)).^22,23,40^ The formation of K_2_O (eqn (9)), K_2_O_2_ (eqn (10)) and KO_2_ (eqn (11)) releases high reaction enthalpies. The oxidation reactions most probably result in the formation of KO_2_ for conditions with high availability of oxygen, due to the higher lattice stability compared to K_2_O and K_2_O_2_.^22,23^

The mass gain of 2.8 ± 0.7 wt.% corresponds to the O_2_ uptake of 0.87 mmol g^−1^, which would require a potassium amount of 0.87 mmol g^−1^ according to eqn (11). This corresponds to 14% of the total potassium, present at the carbon nanofibre samples, which was determined *via* ICP-OES (S2).† The calculated reaction heat of the formation of 0.87 mmol g^−1^ KO_2_ is −248 J g^−1^ according to eqn (11), which is sufficient to cause a significant temperature rise.

However, these reactions cannot explain the subsequent mass loss of 3.1 ± 1.1 wt.%. An analysis of the gaseous products determined CO_2_ as main emission (S3),† suggesting a carbon partial combustion reaction caused by the reaction heat of the potassium oxidation. A total carbon loss of 2.62 mmol g^−1^ is calculated *via*eqn (12) accompanied by −1020 J g^−1^ reaction heat. In total potassium oxidation and carbon combustion reactions result in an emitted reaction heat of −1268 J g^−1^.

12 C + O_2_ ↔ CO_2_, Δ*H*_R_ = −393 kJ mol^−1^

Comparing the calculated reaction heats of KO_2_ and CO_2_ formation, the emitted reaction heat is caused to almost 80% by the carbon combustion to CO_2_. According to the basic heat equation of thermodynamics, the emitted heat could cause a temperature increase to a temperature higher than 700 °C, which would explain the observed glowing of the carbon nanofibres (Fig. 1).

Based on the calculated carbon loss the theoretical released amount of CO_2_ would be 2.58 mmol g^−1^, which equals a CO_2_ release of 114 mg g^−1^. The amount of CO_2_ determined *via* quantification of the gaseous products is lower, giving a value of 40.5 mg g^−1^. The significant difference could be explained by the emission of solid particles due to the vigorous reaction, which are not detected by the MS. Additionally, side reactions due to formed KO_2_ are possible during the reaction as listed in eqn (13) and (14). The possible side reactions mainly result in the formation of potassium carbonate and bicarbonate.^41,42^

13 2KO_2_ + CO_2_ ↔ K_2_CO_3_ + 1.5O_2_, Δ*H*_R_ = −186 kJ mol^−1^

14 2KO_2_ + H_2_O + 2CO_2_ ↔ 2KHCO_3_ + 1.5O_2_, Δ*H*_R_ = −328 kJ mol^−1^

Furthermore, combined TG-DSC measurements were conducted to obtain experimental data on the emitted reaction heats for high O_2_-flow (Fig. 3b). The simultaneous TG measurement was done to assess the comparability of both measurements since different sized crucibles had to be used for both measurements. An emitted reaction heat of −2717 J g^−1^ was determined for the post-treatment step at high O_2_-flow (Fig. 3b). This value is about twice as high as the calculated reaction heat of −1268 J g^−1^ from previous TG measurements (Fig. 3a).

The deviation of these two methods is explicable, when considering the shape of the TG signal of the DSC measurement. Similar to the first discussed TG signal, a mass gain is observed directly after the switch to the high O_2_-flow. Subsequently, a significant mass loss due to carbon combustion occurs. However, the mass loss during the DSC measurement is 8.2 wt.%, which is more than double the value of 3.1 wt.% during the TG measurement. Due to the increased carbon combustion during the DSC measurement the obtained experimental value and the calculated reaction enthalpy from the TG measurement may not be directly comparable. The increased carbon combustion is probably caused by denser packed carbon nanofibres in the smaller DSC crucible, resulting in a stronger heat accumulation. This is an important hint, as the extent of potassium oxidation and carbon combustion reactions depend on the packing density and, therefore, heat and oxygen transport properties of the material.

Interestingly, similar observations are not described for KOH activation of commonly used carbon precursors, *e.g.* PAN powder,^43,44^ carbon fibres,^45–47^ biomass^48–50^ and coals.^18,51,52^ Although, the occurrence of these potassium oxidation reactions could be expected based on the proposed mechanisms for KOH activation mentioned in eqn (6) and (7). Therefore, PAN powder was used as reference material and activated using the identical conditions as for activation of the nanofibre material. During high O_2_-flow post-treatment no pyrophoric effect was observed (S4).† Hence, the described pyrophoric effect is probably linked to the material structure induced by the electrospinning process and, possibly, the activation conditions.

Overall, the post-treatment at high O_2_-flow conditions results in the uncontrolled oxidation of metallic potassium to KO_2_ causing a carbon combustion under severe formation of heat and a strong glowing of the carbon nanofibres.

### 3.2 Low O_2_-flow

**Fig. 4:**
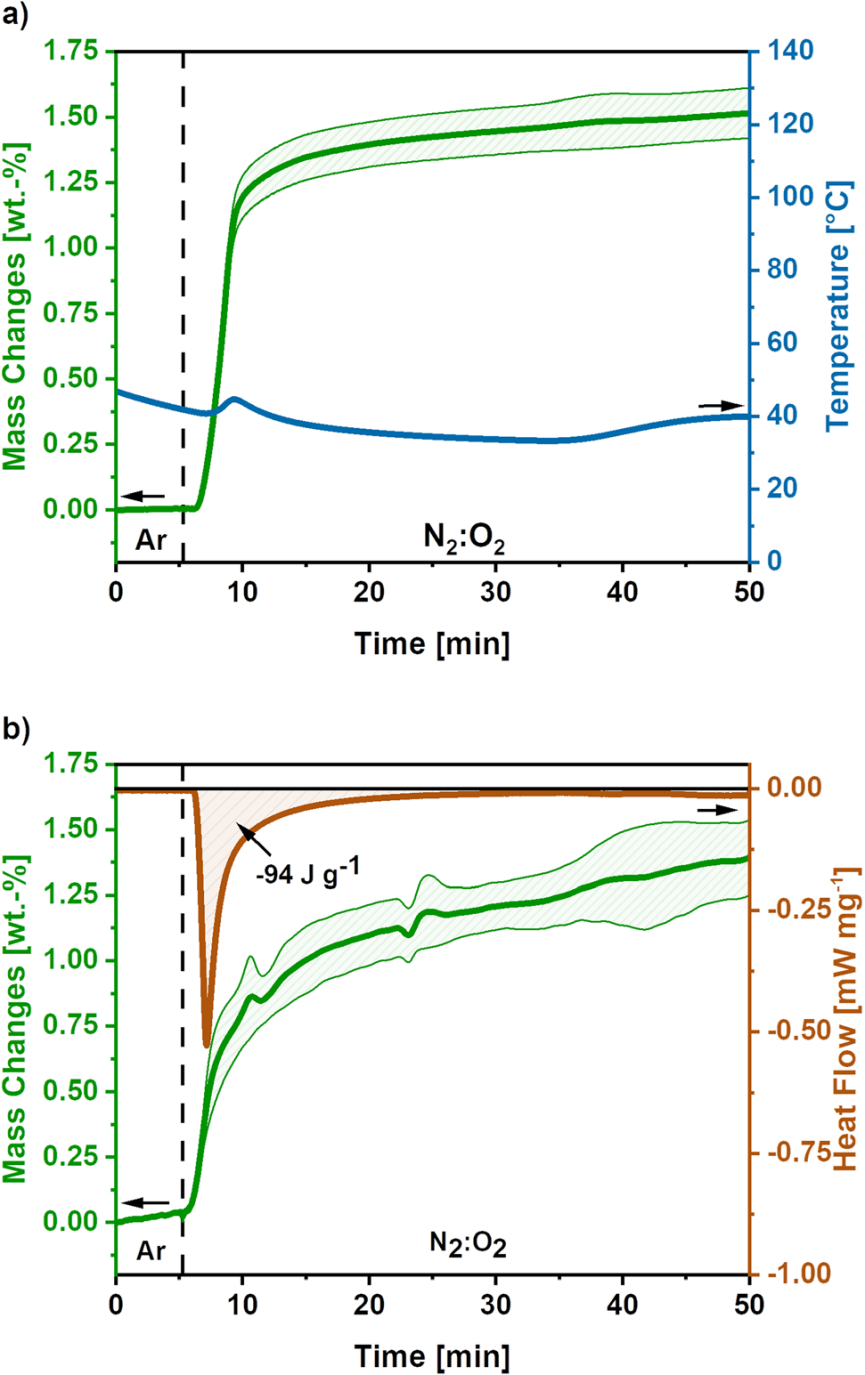
(a) Mass and temperature changes after the switch to low O_2_-flow (4 mL min^−1^) obtained as mean value of 5 measurements. The dashed line indicates the switch from Ar to N_2_ : O_2_ atmosphere and the shaded areas show the standard deviation for the mass signal. (b) DSC-TGA signal for low O_2_-flow post-treatment of activated carbon nanofibres based on 3 measurements. The dashed line indicates the switch to N_2_ : O_2_ atmosphere and the shaded areas show the standard deviation. After the switch a mass gain of 1.5 wt.% is observed accompanied by an emitted reaction heat of −94 J g^−1^.

To develop a treatment strategy to suppress the sample glowing, similar experiments were conducted using an O_2_-flow of 4 mL min^−1^, ‘low O_2_-flow’. Fig. 4a shows the mass and temperature changes after the switch of atmosphere from Ar to a mixture of N_2_ : O_2_ (80 : 20) with an O_2_-flow rate of 4 mL min^−1^, indicated by the dashed line.

A mass gain of 1.5 ± 0.1 wt.% accompanied by a minor temperature rise to 45 °C after 8 minutes is observed after the atmosphere switch. Contrary to the high O_2_-flow, no weight loss is observed. The mass gain can be explained by the uptake of O_2_ due to the oxidation of metallic potassium according to eqn (9)–(11). As the temperature increase is insufficient to cause a carbon combustion, no mass loss is observed.

The obtained mass gain equals an O_2_ uptake of 0.44 mmol g^−1^, based on eqn (12) this results in the emission of −128 J g^−1^ reaction heat. This calculated reaction heat is comparable to the experimental value of −94 J g^−1^ obtained from additional DSC measurements (Fig. 4b) and causes a slight temperature rise on the carbon nanofibres. The slight deviation of the obtained reaction heats can be explained by the simple nature of the calculations used, which only considered one potassium formation reaction neglecting possible side reactions to the formation of other potassium oxide species.^22,23,41^

Summarising, the low O_2_-flow post-treatment results in the controlled oxidation reactions during exposure to oxygen. The emitted reaction heat of −94 J g^−1^ is significantly reduced compared to −2717 J g^−1^ for high O_2_-flow. Therefore, no indication for a carbon combustion or glowing was found.

### 3.3 Comparison of high O_2_-flow and low O_2_-flow

Besides the previously discussed mass and temperature changes during the sample treatment with high O_2_-flow and low O_2_-flow, this section addresses differences in the emitted CO_2_ amount, elemental composition and morphological changes observed in SEM.

#### 3.3.1 Mass balances

**Fig. 5:**
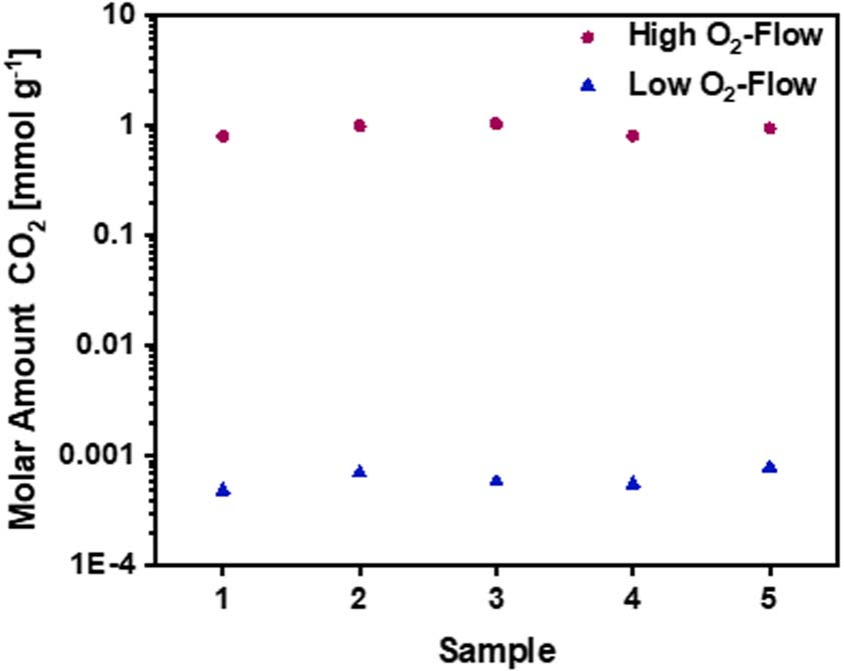
Molar amounts of CO_2_ for high O_2_-flow and low O_2_-flow samples during post-treatment. Data obtained from MS measurements using a CaC_2_O_4_ × H_2_O calibration. A CO_2_ amount of 1 mmol g^−1^ for high O_2_-flow compared to 0.6 μmol g^−1^ for low O_2_-flow was detected.

Fig. 5 shows the emitted amount of CO_2_ in mmol g^−1^ for high O_2_-flow and low O_2_-flow as measured by the MS. The detected CO_2_ amount for high O_2_-flow is at 1000 μmol g^−1^, whereas the CO_2_ emission for low O_2_-flow was at 0.6 μmol g^−1^. This means a reduction of the emitted CO_2_ amount of more than three orders of magnitude for low O_2_-flow conditions. These results match with the absence of a mass loss for low O_2_-flow discussed in the previous section.

Additionally, CHNO elemental analysis was conducted to detect changes in the elemental composition of the obtained carbon nanofibres for high and low O_2_-flow (Table 1). The C content for low O_2_-flow is 69.6 wt.%, whereas it is at 59.8 wt.% for high O_2_-flow. This 10 wt.% decreased C content can be related to the carbon combustion and possibly also to oxidation of the carbon surface. For the H and N content no significant changes were obtained between high O_2_-flow and low O_2_-flow. Regarding the O content, an increase of 7 wt.% for high O_2_-flow compared to low O_2_-flow is observed. This is explained by the reduced amount of C relative to the O content and a partial oxidation of the carbon surface. However, the oxidation of the carbon surface would counteract the observed weight loss during the carbon combustion.

**Table 1:** CHNO elemental composition for neutralized high O_2_-flow, low O_2_-flow and pristine carbon nanofibres. O content is calculated as difference for high O_2_-flow and low O_2_-flow. Elemental composition of the pristine carbon nanofibres taken from.^30^ The value is the average of 5 measurements, the standard deviation is given as error

Sample	Elemental composition (wt%)			
	C	H	N	O
High O_2_-flow	59.8 ± 1	2.2 ± 0.4	12.7 ± 0.5	25.3 ± 3.1
Low O_2_-flow	69.6 ± 0.9	1.7 ± 0.2	10.7 ± 0.6	18.0 ± 2.8
Pristine fibres	72.6 ± 0.1	1.4 ± 0.1	16.2 ± 0.2	9.8 ± 0.7

For further structural characterization and comparison, Raman spectroscopy was performed on low and high O_2_ flow samples. The results are shown and discussed in the ESI (S5).†

#### 3.3.2 Electron microscopy

**Fig. 6:**
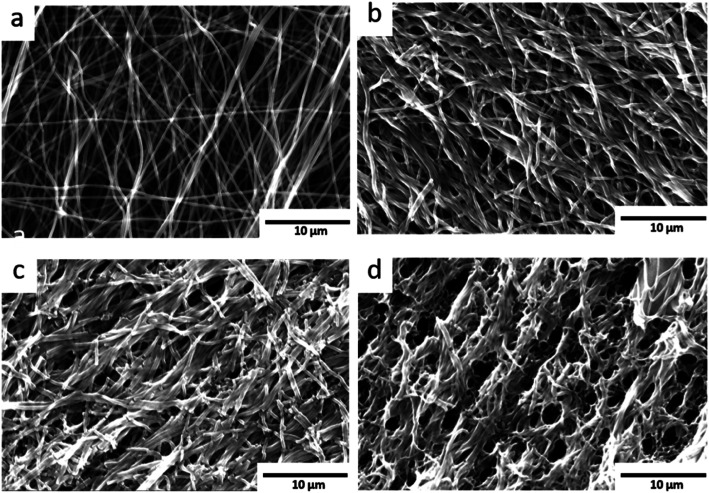
SEM images: (a) pristine carbon nanofibres shown as reference. (b) Low O_2_-flow samples without visible destruction of the fibre morphology. (c) High O_2_-flow samples with an area displaying a smaller degree of destruction (d) high O_2_-flow samples with severe destruction of the fibre morphology.

As the described glowing and combustion effect on high O_2_-flow might cause severe changes to the surface morphology of the carbon nanofibres, high O_2_-flow and low O_2_-flow samples were investigated with SEM (Fig. 6). For reference purposes the pristine fibres^30^ are shown in Fig. 6a as well.

The pristine carbon nanofibres are randomly aligned and large void volumes are visible between the fibres. For the KOH activated carbon nanofibres smaller void volumes and fragmented fibres are observed. The low O_2_-flow samples exhibit a similar morphology as the pristine material (Fig. 6b, S6†*vs.*6a). In contrast, high O_2_-flow samples (Fig. 6d) exhibit areas with significant destruction of surface morphology compared to the pristine material (Fig. 6a). The formation of voids in the size of up to 3 microns is clearly visible in the SEM images (Fig. 6d). These macropores are not homogeneously distributed on the surface of the high O_2_-flow samples as also areas with less severe destruction are visible (Fig. 6c). This observation could be explained by an inhomogeneous distribution of metallic potassium on the samples and, therefore, inhomogeneous severe heat formation due to the potassium oxidation reactions.

Summarising, the obtained SEM results prove that the control of post-treatment conditions significantly affects the morphology of the samples. The applied O_2_-flow affects the potassium oxidation reactions and a severe destruction of the carbon nanofibres can be avoided by application of a low O_2_-flow as post-treatment.

#### 3.3.3 Gas adsorption properties

To obtain detailed information on the impact of the different post-treatment conditions on the pore structure and the adsorption properties, gas adsorption measurements were conducted for high O_2_-flow and low O_2_-flow samples.

**Fig. 7:**
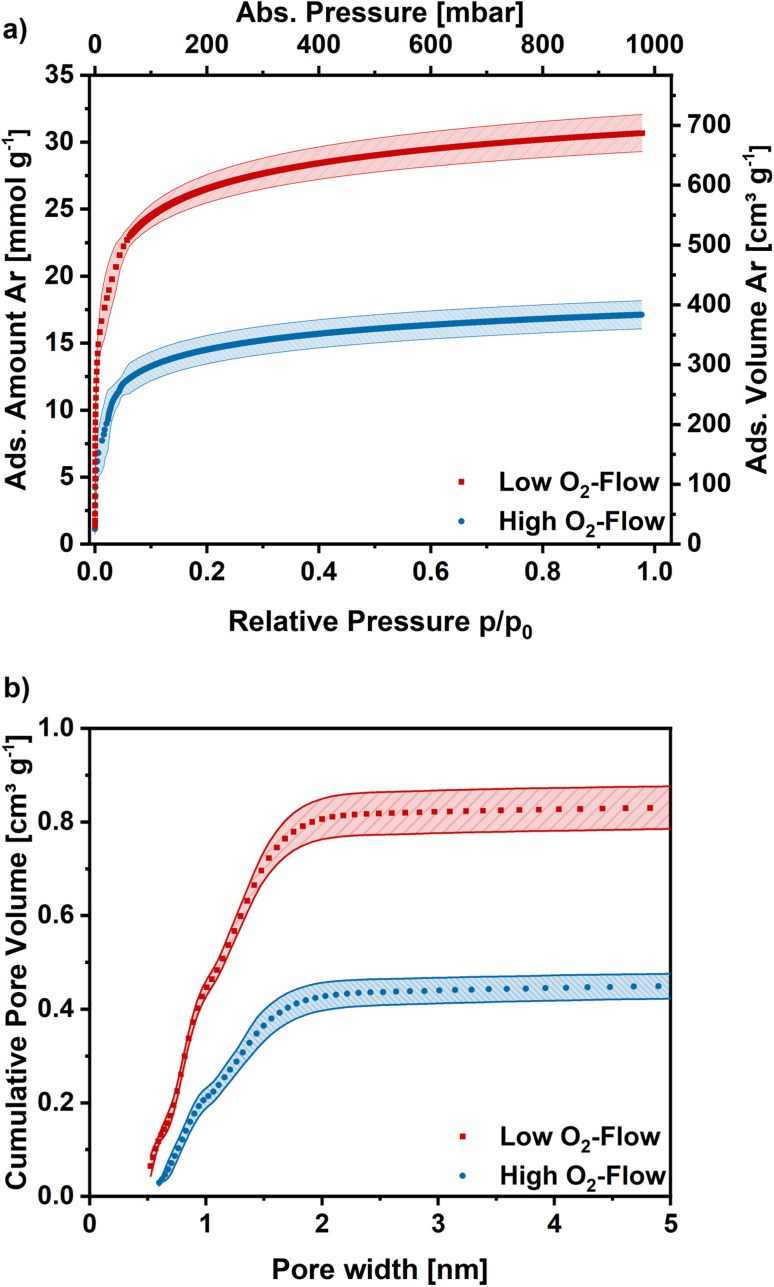
(a) Ar adsorption isotherms obtained at 87 K for high O_2_-flow and low O_2_-flow. Average isotherm of 5 measurements. (b) Cumulative pore size distribution for high O_2_-flow and low O_2_-flow calculated with a DFT kernel. Shaded areas show the standard deviation.

Argon adsorption measurements were performed at 87 K to assess the microporosity. The obtained adsorption isotherms exhibit a type I shape, which is typical for highly microporous adsorbents (Fig. 7a). In the low relative pressure range a steep increase is observed, which flattens for higher relative pressures and approaches a limiting value. The obtained isotherms exhibit similar shapes independent of post-treatment, although the obtained adsorption capacities are significantly higher on the low O_2_-flow samples. At 1 bar 17 mmol g^−1^ CO_2_ are adsorbed on the low O_2_-flow samples, whereas 31 mmol g^−1^ CO_2_ are obtained for high O_2_-flow. This equals an increase in adsorption capacity at 1 bar by 82%.

The pore size distribution was derived from the Ar adsorption isotherms using a density functional theory (DFT) kernel. The cumulative pore size distributions for low O_2_-flow and high O_2_-flow are shown in Fig. 7b. The comparison of the total pore volume *V*_DFT(tot)_ and the pore volume of pores below 2 nm (*V*_DFT<2nm_) reveals that both samples have almost exclusively micropores (Table 2, *V*_DFT<2nm_). The obtained micropore volume is 0.806 cm^3^ g^−1^ for low O_2_-flow samples, which is an increase of 90% compared to 0.424 cm^3^ g^−1^ for the high O_2_-flow. Such values are comparable to values obtained by Im *et al.* and Chiang *et al.* for PAN-based activated carbon fibres.^45,53^

The BET area was determined at 1094 m^2^ g^−1^ for high O_2_-flow and at 2029 m^2^ g^−1^ for low O_2_-flow, which are typical BET areas of KOH-activated carbons.^9,44,54^ Especially the direct comparison to the BET area of the pristine fibres (13.4 m^2^ g^−1^) clearly proves a successful KOH activation of the electrospun PAN-based carbon nanofibres. Additionally, the high impact of the applied post-treatment conditions on the surface morphology and porosity of the carbon nanofibres is obvious.

**Fig. 8:**
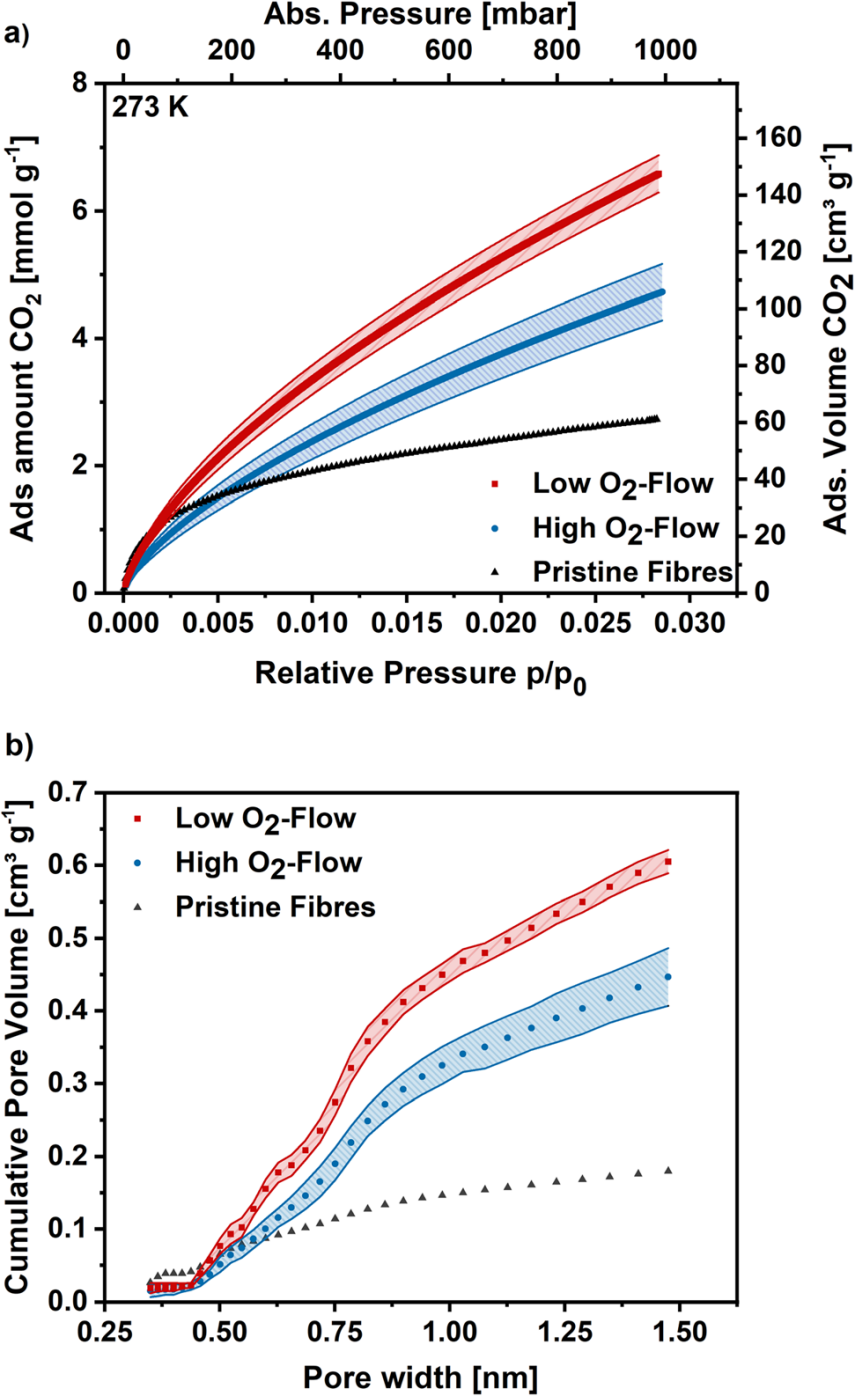
(a) CO_2_ adsorption isotherms at 273 K for high O_2_-flow, low O_2_-flow and the pristine fibres. (b) Determined pore size distribution *via* Monte-Carlo calculations for pores smaller 1.5 nm.

For more detailed micropore characterization CO_2_ adsorption measurements at 273 K were performed. Fig. 8a shows the CO_2_ adsorption isotherms for the pristine,^30^ high O_2_-flow and the low O_2_-flow samples. The pristine fibres isotherm exhibits a high uptake at low relative pressures and turns more into a linear shape at higher relative pressures with a maximum CO_2_ uptake of 2.7 mmol g^−1^ at 1 bar. In comparison, the adsorption isotherm of the high O_2_-flow shows a lower CO_2_ uptake at pressures below 200 mbar.

**Table 2:** BET area, pore volume calculated by DFT calculations (*V*_DFT_) and pore volume calculated using a Monte-Carlo method (*V*_MC_) for low O_2_-flow, high O_2_-flow and the pristine fibres obtained from gas adsorption isotherms of Ar and CO_2_

Sample	BET (m^2^ g^−1^)	*V*_DFT(tot)_ (cm^3^ g^−1^)	*V*_DFT<2nm_ (cm^3^ g^−1^)	*V*_DFT<1.5nm_ (cm^3^ g^−1^)	*V*_MC(tot)_ (cm^3^ g^−1^)	*V*_DFT<0.7nm_ (cm^3^ g^−1^)	*V*_MC<0.7nm_ (cm^3^ g^−1^)
Low O_2_-flow	2029	0.831	0.806	0.696	0.605	0.173	0.208
High O_2_-flow	1094	0.461	0.424	0.365	0.446	0.058	0.145
Pristine fibres	13.4	0.016	0.004	0.003	0.180	—	0.101

At pressures above 200 mbar the adsorption isotherm of CO_2_ surpasses the adsorption isotherm of the pristine fibres and rises to 4.7 mmol g^−1^ at 1 bar (Fig. 8). Contrary, the low O_2_-flow samples exhibit a similar uptake as the pristine fibres at low relative pressures and already surpasses the adsorption capacity of the pristine fibres at pressures of 75 mbar. At 1 bar it reaches an CO_2_ adsorption capacity of 6.5 mmol g^−1^, which is 38% increase compared to the high O_2_-flow samples and even 140% increase compared to the pristine fibres.

In comparison to literature data the obtained CO_2_ adsorption capacities are among the highest for KOH-activated electrospun carbon nanofibres. Wang *et al.* reported CO_2_ adsorption capacities of 2.9 mmol g^−1^ at 1 bar and 273 K, which is significantly lower than the reported values in the present work.^47^ Comparable CO_2_ adsorption capacities were reported by Chiang *et al.* and Zainab *et al.* who obtained 3.5 mmol g^−1^ at 298 K, which is close to the obtained 4.2 mmol g^−1^ at 293 K on the high O_2_-flow sample in this study (S6).† ^55,56^

The pore size distribution was determined using Monte-Carlo calculations and the cumulative pore volume is shown in Fig. 8b. As described previously for DFT calculations based on Ar adsorption, the pore volume increases for low O_2_-flow samples over the full range of pore sizes compared to the high O_2_-flow samples. The total pore volume is 0.61 cm^3^ g^−1^ for low O_2_-flow and 0.45 cm^3^ g^−1^ for high O_2_-flow samples, resulting in an increase in pore volume of 36% for the low O_2_-flow samples. Regarding the ultramicropore volume (<0.7 nm), high O_2_-flow samples exhibit a value of 0.15 cm^3^ g^−1^, which is enhanced to 0.21 cm^3^ g^−1^ on low O_2_-flow samples. Overall, the obtained pore volumes from CO_2_ adsorption isotherms are comparable to those obtained from Argon isotherms, except for a significant difference of the obtained micropore volume below 1.5 nm for DFT (0.365 cm^3^ g^−1^) and Monte-Carlo (0.446 cm^3^ g^−1^) data for high O_2_-flow. This deviation could be caused by the different surface chemistry of samples prepared at high and low O_2_-flow. High O_2_-flow samples exhibit a higher oxygen content than low O_2_-flow samples, which could affect the interactions of adsorptive and adsorbent. Such effects are not accounted for in the standard calculation models used for the determination of the pore size distributions.

**Fig. 9:**
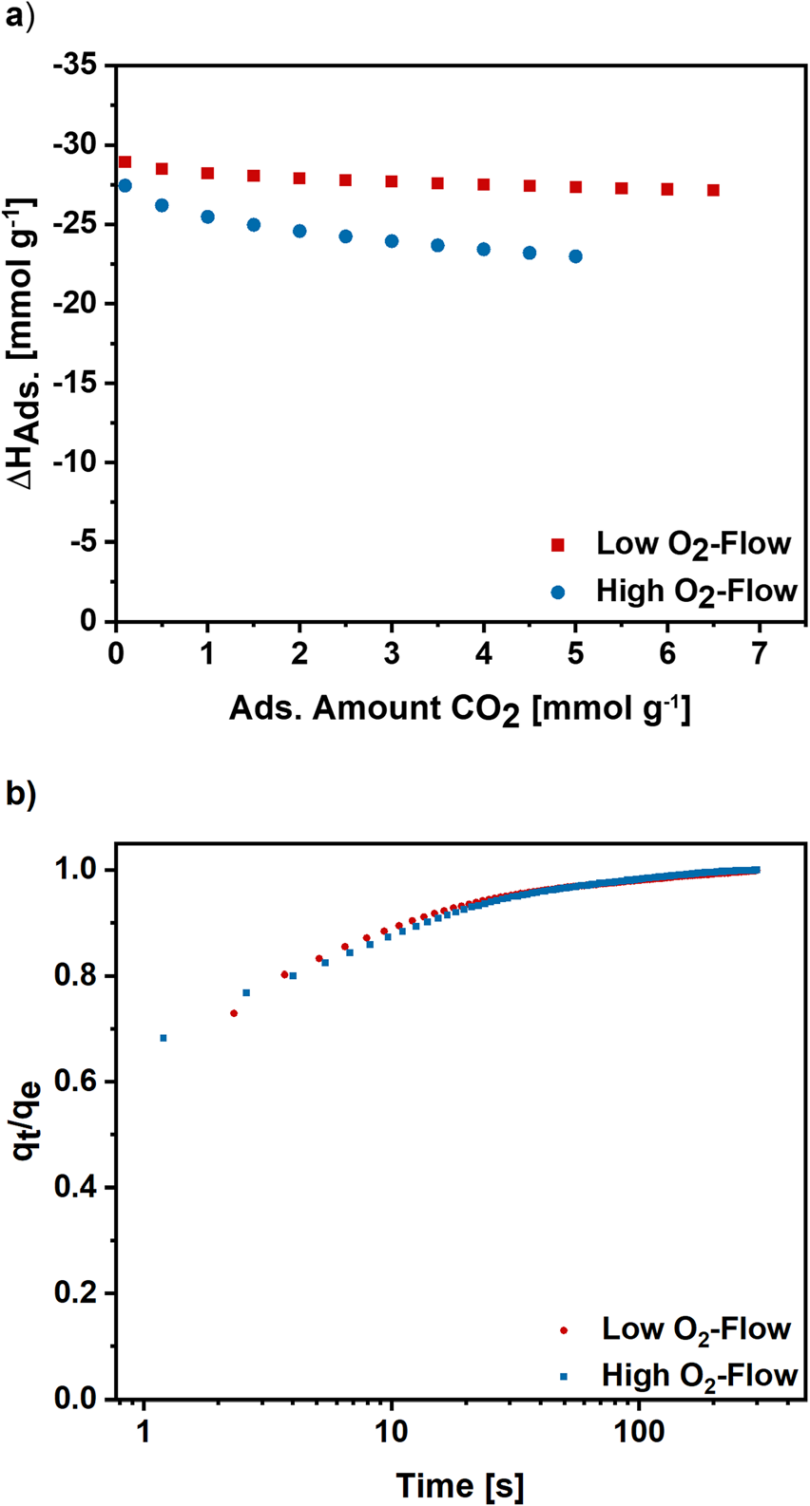
(a) Isosteric enthalpy of adsorption for high and low O_2_-flow calculated for CO_2_ isotherms measured at 273 K, 283 K and 293 K. (b) Normalized adsorption kinetics for high and low O_2_-flow determined at 50 mbar and 298 K.

Furthermore, the isosteric enthalpy of adsorption was calculated for high and low O_2_-flow based on CO_2_ adsorption isotherms measured at 273 K, 283 K and 293 K (S7).† For low O_2_-flow a value of 28.9 kJ mol^−1^ was determined at a loading of 0.1 mmol g^−1^ which slightly decreases to 27.1 kJ mol^−1^ at a loading of 6.5 mmol g^−1^ (Fig. 9). For high O_2_-flow, the obtained enthalpy of adsorption is at 27.4 kJ mol^−1^ at a loading of 0.1 mmol g^−1^ and decreases to 23.0 kJ mol^−1^ at a loading of 5 mmol g^−1^. Both isosteric enthalpies of adsorption are comparable to adsorption enthalpies of activated electrospun carbon nanofibres in literature.^57^ The more distinctive decrease of the isosteric enthalpy of adsorption for high O_2_-flow can be linked to a higher degree of surface oxygen due to the partial carbon oxidation at high O_2_-flow (Table 1), which probably lowers the binding affinity towards CO_2_ due to the increased number of acidic groups on the surface.

To get an insight into the adsorption kinetics, equilibration curves of CO_2_ at 50 mbar and 298 K were measured (Fig. 9b). For high and low O_2_-flow the adsorption rate is fast, as the equilibrium loading is reached within the first 100 s with the steepest increase in the first 20 s. Comparing the two post-treatments, there are no significant changes of the adsorption kinetics. Therefore, the choice of post-treatment does not notably affect the adsorption rate of the carbon nanofibres.

Overall, the low O_2_-flow conditions result in improved gas adsorption properties, namely a higher adsorption capacity and a higher micropore volume. Furthermore, the isosteric enthalpy of adsorption is increased for low O_2_-flow, whereas the adsorption kinetics are very similar for both post-treatments. Based on the results, the importance the choice of post-treatment conditions after KOH activation of electrospun carbon nanofibres becomes evident.

## 4. Conclusion

The effect of two atmospheric post-treatments after the KOH activation of electrospun PAN-based carbon nanofibers was detailed studied with a focus on the occurring pyrophoric effect, chemical reactions during the post-treatment and changes of the morphology and adsorption properties.

At high O_2_-flow conditions a significant formation of heat was observed and related to the oxidation reactions of metallic potassium, which was formed during the activation process prior to the post-treatment step. The reaction heat of the potassium oxidation reactions acts as igniter for a subsequent carbon combustion, which significantly changes the pore structure and surface chemistry of the material and destroys the original fibre structure. The comparability of high O_2_-flow to a usually applied ambient air post-treatment was shown in an additional experiment in which a significant pyrophoric effect was visible.

Control of the vigorous potassium oxidation reactions was enabled by the application of a low O_2_-flow as post-treatment. The low O_2_-flow limits the oxidation reactions, resulting in a reduced heat formation, which is insufficient to cause a carbon combustion. A significant increase in adsorption capacities and accessible pore volume as well as higher enthalpies of adsorption of CO_2_ were found for low O_2_-flow samples.

Summarising, this work clearly shows that vigorous potassium oxidation reactions can occur after the KOH activation of electrospun carbon nanofibres and alter the obtained material. By proper choice of the atmospheric post-treatment conditions this potassium oxidation reactions can be limited and a significant improvement of the obtained porosity and surface chemistry can be achieved.

## Data availability

Data will be made available upon reasonable request.

## Conflicts of interest

The authors have no competing interest to declare.

## Supplementary Material

RA-014-D3RA07096D-s001
