# Supplementary material for: Post-treatment strategies for pyrophoric KOH-activated carbon nanofibres
Source: RSC Adv. 2024 Jan 25;14(6):3845–56. doi: 10.1039/d3ra07096d (PMC10810230; doi:10.1039/d3ra07096d)
Supplement: RA-014-D3RA07096D-s001 [file RA-014-D3RA07096D-s001.pdf]

Electronic Supplementary Material (ESI) for RSC Advances.
This journal is © The Royal Society of Chemistry 2024

Post-Treatment Strategies for Pyrophoric Electrospun PAN-based Carbon Nanofibers

T. Fischer[a,b,*], A. Kretzschmar[a], V. Selmert[a,b], S. Jovanovic[a], H. Kungl[a], H. Tempel[a], R-A. Eichel[a,b]

[a]Forschungszentrum Jülich GmbH, Institute of Energy and Climate Research (IEK-9) – Fundamental Electrochemistry, 52425 Jülich, Germany
[b]RWTH Aachen University, Institute of Physical Chemistry, 52056 Aachen, Germany

Supporting Information

S1. Calibration data used for determination of the emitted molar amount of CO_2

50, 75, 100, 125 and 150 mg of $CaC_2O_4 * H_2O$ were heated to 1000 °C inside the TGA-MS. Measurement run in MID mode. Determination of the peak area of m/z 44 during the decomposition of formed $CaCO_3$ into CaO and CO_2. Based on the fragmentation data from NIST database the total peak area due to CO_2 was determined and used as calibration data.

$$CaC_2O_4 * H_2O \leftrightarrow CaC_2O_4 + H_2O\ (>150\ °C)$$

$$CaC_2O_4 \leftrightarrow CaCO_3 + CO\ (>500\ °C)$$

$$CaCO_3 \leftrightarrow CaO + CO_2\ (T > 750\ °C)$$

Table 1: Relative Intensities of the ion fragmentation of CO_2 in mass spectrometry for m/z 44, 28, 16 and 12.

Ion Fragmentation CO_2			
m/z 44	m/z 28	m/z 16	m/z 12
1	0.098	0.097	0.085

(Peter Linstrom (2017), NIST Chemistry WebBook - SRD 69, National Institute of Standards and Technology, https://doi.org/10.18434/T4D303 (Accessed 2023-03-01).

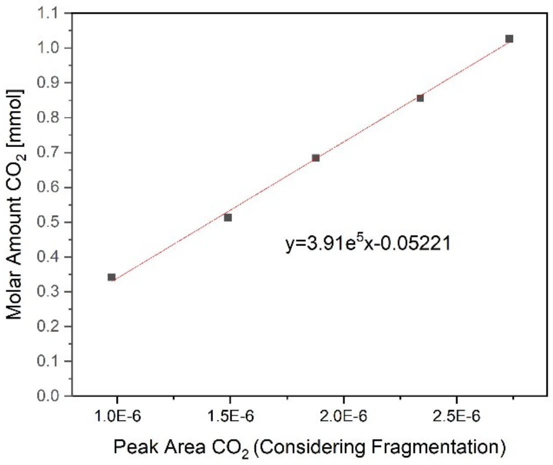

Figure 1: MS Calibration data obtained from $CaC_2O4_4*H_2O$ calibration. 50, 75, 100, 125 and 150 mg $CaC_2O_4*H_2O$ were heated up to 1000 °C inside the TGA-MS. A Relation between the determined peak area and the molar amount of CO_2 can be drawn.

S2. ICP-OES Results

Table 2: Determination of the total potassium content of impregnated, Low O_2-Flow and High O_2-Flow carbon nanofibers.

Sample	K [wt.-%]	Std. Dev.
**After KOH impregnation**	25.2	0.6
**Low O_2-Flow before neutralization**	26.8	0.7
**High O_2-Flow before neutralization**	30.4	0.4

S3. Mass spectrometer scan measurement with m/z = 0-100 for the detection of gaseous reaction products

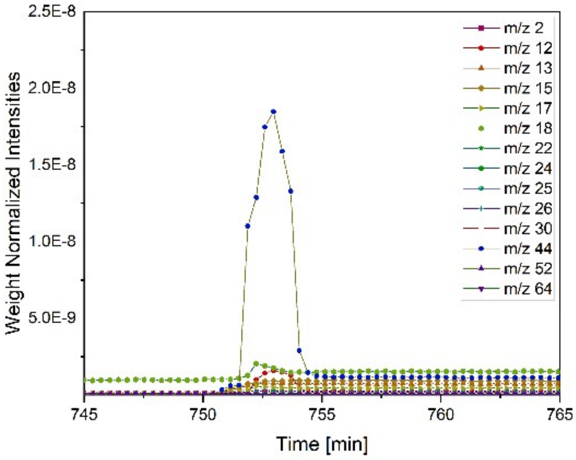

Figure 2: MS Scan m/z 0-100 prior to the multiple ion detection runs to detect significant gaseous products. Measurements were obtained during atmospheric post-treatment at High O_2-Flow conditions after the simultaneous carbonization and activation of electrospun PAN-based carbon nanofibers. M/Z 44 (CO_2) detected as main emission.

S4. Reference Material – PAN Powder

Stabilized PAN powder was used to investigate, whether a pyrophoric effect is also observed for a typical reference material. The used experimental parameters are listed below:

Stabilized PAN-Powder:
PAN powder (150.000 g mol^{-1}, BOC Science, USA) was stabilized at 250 °C for 15 hours in air.

Activation process:
The stabilized PAN powder was impregnated and activated using fully identical conditions to the given description in the paper. 200 mg of the powder material were impregnated with 10 ml of 0.26 mol L^{-1} KOH solution. The activation process was conducted inside the TGA-MS (STA 449 F1 Jupiter, Netzsch GmbH, Germany) at a heating rate of 300 K h^{-1} and the sample was carbonized and activated for 3 hours in Ar 5.2 atmosphere. After cooling down at a rate of 200 K h^{-1} the sample was exposed to High O_2-flow conditions.

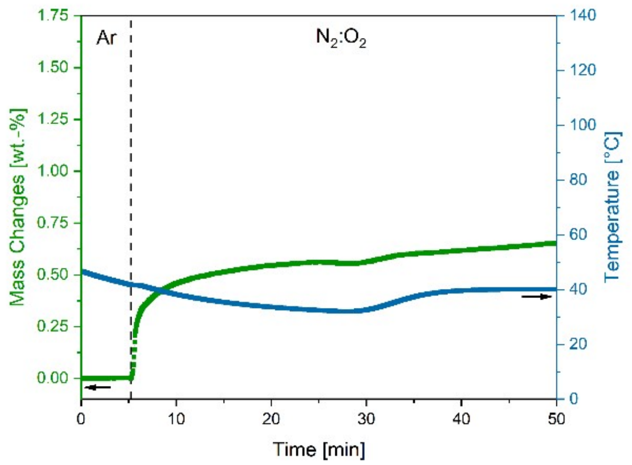

Figure 3: Mass and temperature changes during post-treatment at High O_2-Flow conditions (175 ml min^{-1} O_2). A mass increase is observed after the switch to post-treatment, which is assigned to potassium oxidation reactions. Since no significant temperature increase is observed for the material, no indication for a carbon burn-off is visible.

S5 Raman Measurements

Raman experiments were conducted using a WITec alpha R Raman microscope (Ulm, Germany) using a 50x magnification Zeiss objective and a 532 nm laser at a power of 2mW. For each sample, High and Low O_2-Flow, Raman spectra were acquired for a total of 30 s measurement time at five different, representative surface locations.

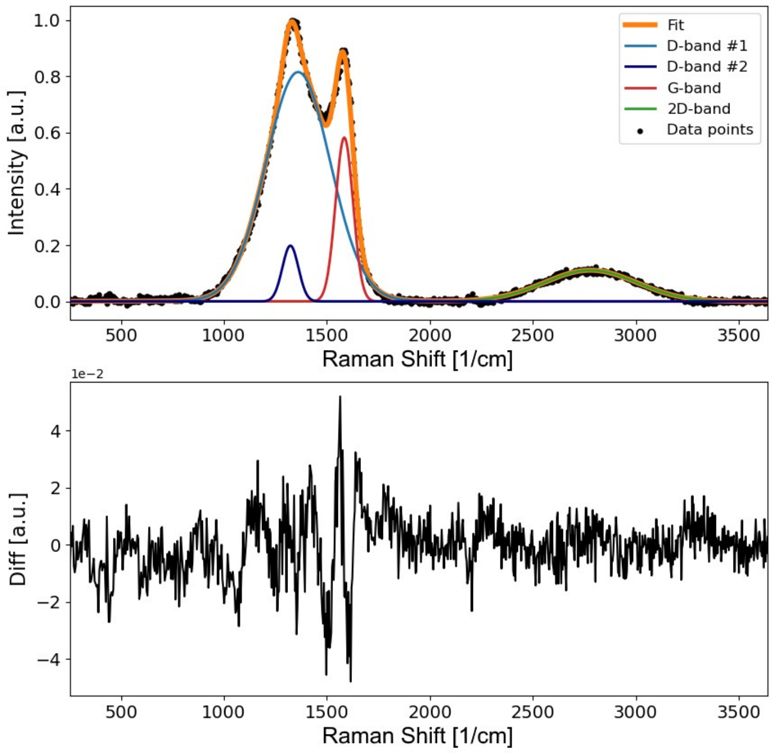

Figure 4: Exemplary fit procedure for determination of the D/G-ratio, shown for a Raman spectrum of a low O_2-flow sample (top). Difference plot between overall fit and data points (bottom), which exhibits only minor systematic errors.

All spectra were baseline corrected using the shape algorithm with shape size 500 in the WITec Control 6.1 software suite (Ulm, Germany). For the determination of the D/G ratio of the overlapping carbon D and G-bands, the spectra were fitted using LMFIT version 0.9.14 for Python[1], employing a non-linear least-squares algorithm. Due to the signal shape, the D-band was fitted using two Gauss peaks, the G-band and 2D-band one Gauss peak, respectively, as shown in figure 7.

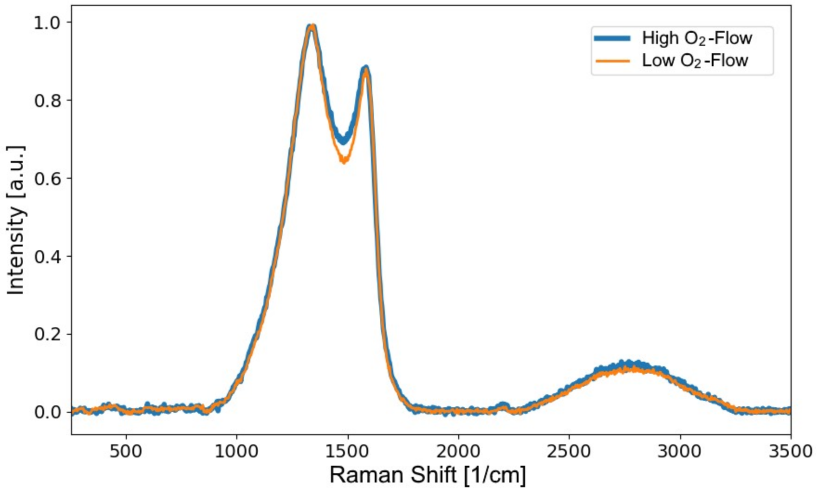

Figure 5: Averaged Raman spectra of high and low O_2-Flow samples. Both samples exhibit a strong D band around 1330 cm^{-1}, a strong G band around 1580 cm^{-1} and a broad 2D band for 2300-3300 cm^{-1}. Only minor differences between the two samples were observed.

Both the low and high O_2-flow carbon fibres exhibit typical Raman spectra (Fig. 8) for amorphous carbon materials. The G-Band at 1580 cm^{-1} is assigned to E_{2g} vibrations of the graphite crystal lattice. The D-band at 1330 cm^{-1} is only observed if defects or disorder are present in the graphite structure. For the carbon fibre materials, the D-band is broad and intense compared to the G-band, indicating the presence of a highly unordered or defect-rich graphite lattice. The broad double-resonance 2D band between 2300 and 3300 cm^{-1} is present for all graphitic independent of lattice defects. In order to qualitatively compare the degree of crystallinity and structural disorder between different carbon compounds, the ratio of D-band to G-band integrals (D/G-ratio) is calculated.
The averaged D/G ratio for the low and high O_2-flow samples is shown in table 4. As is evident from the calculated standard deviation, no significant deviations of Raman signals were observed for the individual measurement spots of each sample. Furthermore, high D/G ratios were determined for both types of carbon fibres. These observations are attributed to the uniform, but highly amorphous carbon structure of the fibres, which is in line with the expectations for a low carbonization temperature of 800 °C. Moreover, no differences in D/G ratio and thus carbon amorphicity were observed within a 3σ (99.6 %) confidence interval. Therefore, no significant differences in carbon structure due to post-treatment could be shown for the two sample types.

Table 3: D/G ratios of carbon fibers post-treated at High and Low O_2-Flow.

**Sample**	**D/G**	**Standard Deviation**
**High O_2-Flow**	5.77	0.0904
**Low O_2-Flow**	5.50	0.1183

1: Newville, M. et al. lmfit/lmfit-py 0.9.14. Zenodo DOI: 10.5281/zenodo.3381550 (2019).

S6. Additional SEM images with different magnifications

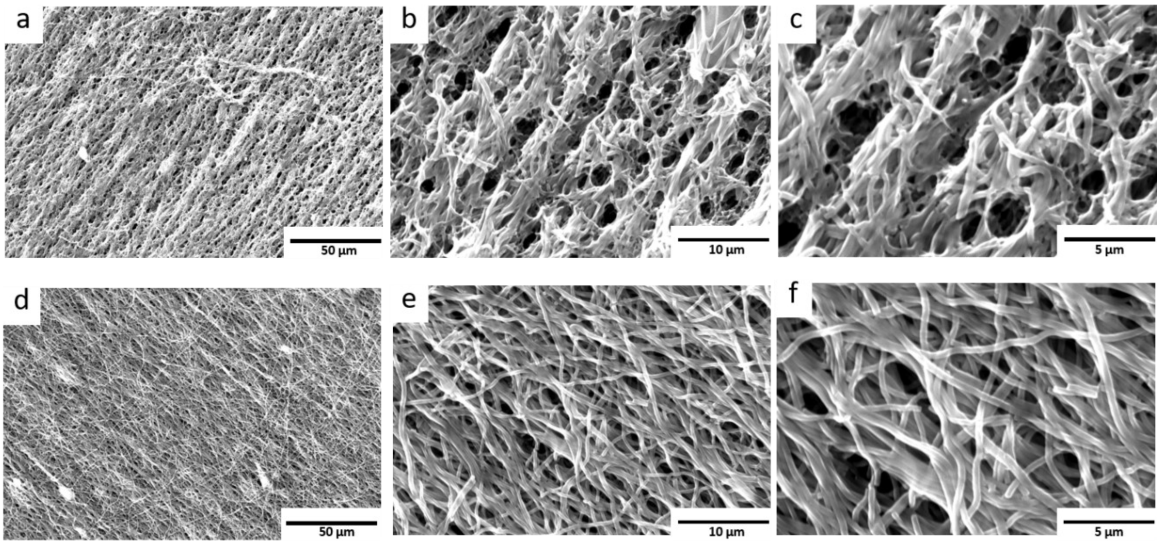

Figure 6: SEM images obtained from neutralized KOH-activated carbon nanofibers after High O_2-Flow (a)-(c) and Low O_2-Flow (d)-(f).

S7. Determination of the isosteric enthalpy heat of adsorption

The isosteric enthalpy heat of adsorption was calculated via the Clausius-Clapeyron approach using CO_2 adsorption isotherms measured at 273 K, 283 K and 293 K. The isotherms were fitted using the Tóth isotherm equation (Eq.1, Table 3).

$$Q_e = Q_{max} * \frac{k * p}{\left(1 + (k * p)^t\right)^{\frac{1}{t}}} \qquad (1)$$

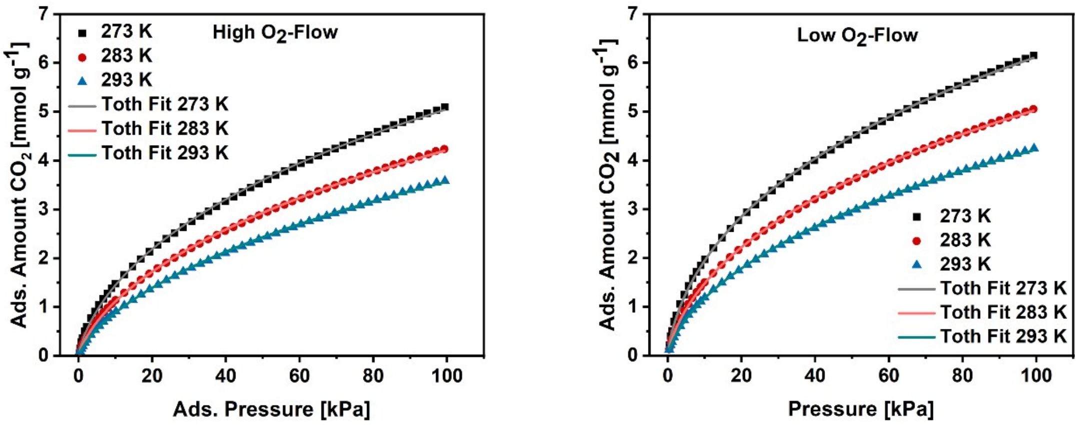

Figure 7: CO_2 adsorption isotherms for High O_2-Flow and Low O_2-Flow samples at 273 K, 283 K and 293 K. Additionally the Tóth Fit isotherm is shown, which was used for the calculation of the isosteric of enthalpy adsorption.

Table 4: Tóth Fit parameters for High O_2-Flow and Low O_2-Flow samples at 273 K, 283 K and 293 K. Q_{max} was limited to below 50 to keep it close to physically realistic values and still obtaining a high R^2. Based on the fit data, the pressures were calculated for the ln p vs. 1/T plots.

	High O_2-Flow			Low O_2-Flow		
	293 K	283 K	273 K	293 K	283 K	273 K
k	0.00668	0.00999	0.01659	0.01332	0.01939	0.03279
Q_{max}	50	50	50	50	50	50
t	0.28497	0.28035	0.27329	0.26538	0.26541	0.26177
R^2	0.99958	0.99964	0.99916	0.99979	0.99985	0.99974

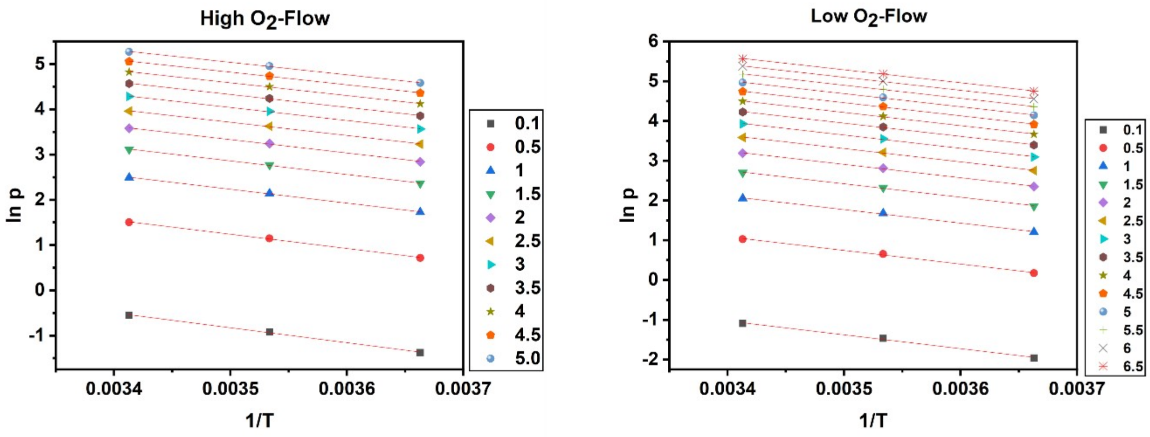

Figure 8: Ln p vs 1/T plots for High O_2-Flow and Low O_2-Flow obtained based on the obtained Toth Fit isotherm data. The obtained slope of the linear graphs for different loadings were used to calculate the isosteric enthalpy of adsorption.
